# Comparison of the Roche cobas® 4800 and Digene Hybrid Capture® 2 HPV tests for primary cervical cancer screening in the HPV FOCAL trial

**DOI:** 10.1186/s12885-015-1959-5

**Published:** 2015-12-16

**Authors:** Darrel A. Cook, Wendy Mei, Laurie W. Smith, Dirk J. van Niekerk, Kathy Ceballos, Eduardo L. Franco, Andrew J. Coldman, Gina S. Ogilvie, Mel Krajden

**Affiliations:** BC Cancer Agency, 675 West 10th Ave, Vancouver, BC V5Z 1L3 Canada; BC Centre for Disease Control, 655 West 12th Ave, Vancouver, BC V5Z 4R4 Canada; Lower Mainland Pathology and Laboratory Medicine, 655 West 12th Ave, Vancouver, BC V5Z 4R4 Canada; University of British Columbia, 2329 West Mall, Vancouver, BC V6T 1Z4 Canada; McGill University, 845 Rue Sherbrooke O, Montréal, QC Canada

**Keywords:** Cervical cancer screening tests, Human papillomavirus DNA tests, Papanicolaou test, Colposcopy triage, Cervical intraepithelial neoplasia

## Abstract

**Background:**

HPV FOCAL is a randomized trial (ISRCTN79347302, registered 20 Apr 2007) comparing high-risk (hr) HPV testing vs. liquid-based cytology (LBC) for cervical cancer screening of women aged 25–65. We compared the Digene Hybrid Capture® 2 High-Risk HPV DNA Test® (HC2) and the Roche cobas® 4800 HPV Test (COBAS) for primary screening.

**Methods:**

Women (*n* = 6,172) were screened at baseline by HC2 and COBAS and by LBC 24 months later. We assessed HPV genotyping and reflex LBC for colposcopy triage of baseline HPV positive women.

**Results:**

Overall HC2/COBAS agreement was 96.1 % (kappa 0.75) and positive agreement was 77.5 %. Baseline CIN2 and CIN3+ rates based on HPV screening were 8.6/1,000 and 6.6/1,000 respectively; 24 month rates were 0.7/1,000 and 0.4/1,000 (LBC screening). HC2 and COBAS were concordant positive for 91 % of round 1 CIN2 and 98 % of CIN3+. CIN3+ was significantly associated with HPV 16 (Odds Ratio [OR] 5.11; 95 % confidence interval [CI] 2.30, 11.37), but not HPV 18 (OR 2.62; 95 % CI 0.73, 9.49), vs. non-HPV 16/18 HPV at baseline. There was no significant association between HPV genotype and CIN2. CIN3+ was significantly more likely for high-grade (OR 5.99; 95 % CI 2.53, 14.18), but not low-grade (OR 0.54; 95 % CI 0.20, 1.49), vs. negative LBC. No significant association was observed between LBC grade and CIN2. HPV 16 and 18 were associated with 33 % of CIN2 and 68 % of CIN3+ identified at baseline.

**Conclusions:**

For hrHPV positive women, abnormal reflex LBC is appropriate for colposcopy triage. In addition, immediate referral of women with HPV 16/18 and normal cytology may allow for earlier detection of CIN2+ lesions which would not be detected until after follow-up testing.

**Electronic supplementary material:**

The online version of this article (doi:10.1186/s12885-015-1959-5) contains supplementary material, which is available to authorized users.

## Background

Persistent infection with high-risk (hr) human papillomavirus (HPV) genotypes is recognized as the cause of cervical cancer [1, 2] and many screening programs are shifting from traditional Pap cytology to hrHPV testing. Screening for hrHPV has higher sensitivity relative to the Pap for detection of cervical precancerous lesions [3] (i.e., cervical intraepithelial neoplasia grade two [CIN2] or CIN2 and worse [CIN2+]). In addition, hrHPV testing has a negative predictive value for CIN2+ of greater than 99 %, enabling screening intervals to be extended up to 5 years or longer [4–6]. A number of hrHPV screening tests are commercially available, many of which incorporate partial genotyping in addition to generating a qualitative positive/negative result [7]. Since HPV 16 and 18 infections account for at least 70 % of cervical cancers worldwide [8], specific detection of these two genotypes provides useful additional information for colposcopy triage of hrHPV positive women who are more likely to have or develop pre-cancerous lesions [9, 10].

A challenge is that hrHPV testing identifies women with both persistent and transient HPV infections, with most of the latter clearing spontaneously within 2 years [11]. For that reason it is undesirable to refer all hrHPV positive women to colposcopy since treatment of lesions which are likely to spontaneously regress is associated with increased health system costs, patient anxiety [12] and the potential for reproductive harms [13, 14]. An efficient triage mechanism is required so that women at the highest risk of developing rapidly progressive cervical disease are referred to colposcopy, whereas those at lower risk could be more closely followed for clearance of transient infections, with colposcopy referral only for those hrHPV infections which persist. Several triage approaches for hrHPV positive women have been assessed, including reflex cytology [5], p16/Ki67 immunostaining [15], HPV 16/18 genotyping [9, 10] and the detection of methylation of HPV and/or human genes [16, 17].

In this study, we compared the performance of the Digene Hybrid Capture® 2 High-Risk HPV DNA Test® (HC2) to that of the Roche cobas® 4800 HPV Test (COBAS) for primary cervical screening within the HPV FOCAL randomized trial. We assessed CIN outcomes following reflex cytology and HPV genotyping for colposcopy triage.

## Methods

### Study population

**Fig. 1:**
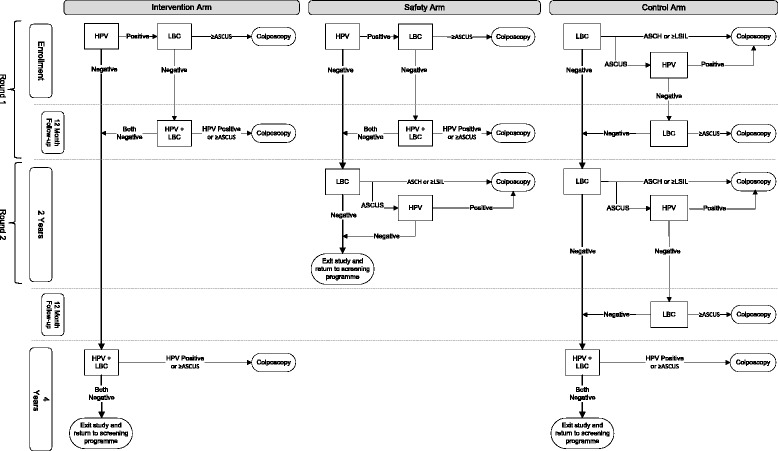
HPV FOCAL Trial Design. *Abbreviations*: *HPV* Digene Hybrid Capture® 2 high-risk HPV DNA test®, *LBC* liquid-based cytology, *ASCUS* atypical squamous cells, undetermined significance, *ASCH* atypical squamous cells, cannot exclude high-grade, *LSIL* low-grade squamous intraepithelial lesion

The HPV FOCAL Trial design has been described previously [18] (Fig. 1). Briefly, the trial is a three-arm, randomized controlled trial (ISRCTN79347302, registered 20 Apr 2007) to establish the efficacy of hrHPV screening (using HC2) with liquid-based cytology (LBC) triage of HC2 positive women (Intervention and Safety Arms) compared to LBC screening with HC2 triage of atypical squamous cells of undetermined significance (ASCUS) (Control Arm) in women aged 25–65 years. The Safety Arm was included to verify the safety of the Intervention Arm 4 year screening interval by re-screening baseline HC2 negative women with LBC at 2 years, comparable to the current standard of care in British Columbia (BC), conventional cytology every 2 years. Women in the Safety Arm are the subjects of the present analysis. At baseline, HC2 positive women with LBC ≥ASCUS were referred immediately to colposcopy, whereas those HC2 positive/LBC negative for intraepithelial lesions or malignancy (NILM) were re-tested by HC2 and LBC at 12 months. Those persistently HC2 positive and/or LBC ≥ASCUS were then referred to colposcopy. Visible lesions were biopsied and if none were seen, endocervical curettage was performed for histological analysis. Baseline HC2 negative women, together with those who cleared hrHPV and were LBC NILM at 12 months, had their Safety Arm exit screen at 24 months. Round 1 refers to the baseline screen together with the 12-month follow-up and Round 2 refers to the exit screen at 24 months (Fig. 1).

All trial subjects provided written informed consent and the study was approved by the University of British Columbia/BC Cancer Agency Clinical Research Ethics Board.

### Laboratory testing

Cervical specimens collected in PreservCyt® (Hologic Inc., Bedford MA, USA) by participating family physicians in Metro Vancouver and Greater Victoria BC were used for both hrHPV testing and LBC. For Safety Arm enrollment and 12 month follow-up specimens, separate aliquots for HC2 (6 mL) and COBAS (2 mL) were removed from the PreservCyt® vial prior to any other processing. At the 24 month exit screen, aliquots for reflex HC2 testing were obtained after cytology processing. At enrollment, all specimens were tested by HC2 (Qiagen, Gaithersburg MD, USA), COBAS (Roche Molecular Systems, Pleasanton CA, USA) and the Roche Linear Array HPV Genotyping Test (LA; Roche Molecular Systems). HC2 generates a qualitative result for the presence or absence of one or more of 13 hrHPV genotypes (HPV 16, 18, 31, 33, 35, 45, 51, 52, 56, 58, 59 and 68). We used a relative light unit/cutoff (RLU/CO) ratio of ≥1.0 to indicate a positive HC2 result. Specimens which had no visible cell pellet at the preparation stage were rejected as inadequate and reported as HC2 invalid. COBAS generates individual qualitative results for HPV 16, HPV 18 and a pool of 12 other hr (OHR) HPV genotypes (HPV 31, 33, 35, 45, 51, 52, 56, 58, 59, 66 and 68). LA generates individual qualitative results for the same 14 hrHPV genotypes as COBAS together with 23 additional HPV genotypes which are defined for the purposes of this study as low-risk (lr) HPV. LA was performed using residual extracts from COBAS testing. COBAS and LA were performed blinded at the time of collection and were not used for subject management. Specimens with initially invalid COBAS results were re-tested to confirm the invalid result. LBC slides were prepared and stained for all subjects at enrollment but were not screened and interpreted unless the subject was HC2 positive. COBAS and LA results were unblinded and compared with HC2 after Safety Arm women exited the trial at 2 years. LBC slides were then screened and interpreted retrospectively for women with baseline HC2 negative/COBAS positive and HC2 negative/LA positive results. For the latter group, specimens positive by LA for at least one hrHPV genotype were classified as hrHPV positive and those positive for only lrHPV genotype(s) were classified as lrHPV positive.

### Statistical analysis

Overall agreement, Cohen’s kappa, and positive and negative agreements with 95 % confidence intervals (CI) were calculated for enrollment HC2 vs. COBAS results. Positive agreement was calculated by the formula *p*_*pos*_ *= (2a)/(N + a - d)* and negative agreement by *p*_*neg*_ *= (2d)/(N – a + d)*, where *N* is the number tested, *a* is the number positive by both tests, and *d* is the number negative by both tests [19]. Genotyping agreements (overall and positive) and Cohen’s kappa with 95 % CI were calculated for COBAS vs. LA.

CIN diagnoses were obtained from the centralized provincial cervical screening registry, which includes all screening and histopathology results for BC women. CIN2 and CIN3+ rates with 95 % CI, stratified by baseline HC2 and COBAS results, per 1,000 women screened were calculated and 95 % CI for rates assumed a Poisson distribution. Round 1 included CIN lesions identified within 2 years after the colposcopy referral date, which could have occurred at baseline or after 12 months follow-up, while round 2 included CIN lesions identified within 2 years of the colposcopy referral date following the exit screen. If a subject had more than one colposcopic biopsy, the result for the highest grade CIN lesion identified was used for analysis. The analyses in this paper were based only on CIN lesions identified among women referred to colposcopy as per the FOCAL trial protocol. For trial quality assurance purposes, periodic audits of the provincial registry were conducted to identify CIN2+ lesions among trial subjects whose colposcopy referrals were not based on the trial protocol. Any additional CIN2+ identified by the audits were not included in the analyses.

A multivariate logistic regression model was fitted to the data to test the hypothesis regarding the relationship between HPV genotype and reflex LBC results and the likelihood of a CIN outcome. Since a given subject may be positive for more than one hrHPV genotype, subjects were classified hierarchically for analysis: for HPV 16, the subject was HPV 16 positive but could also be positive for HPV 18 and OHR HPV genotypes; for HPV 18, the subject was HPV 16 negative, HPV 18 positive, and could also be positive for OHR HPV genotypes; for OHR HPV, the subject was negative for HPV 16 and 18 and positive for one or more of the OHR HPV genotypes.

The Median score and Mann-Whitney U tests were used to calculate p values for the median HC2 RLU/CO and COBAS cycle threshold (Ct) values for concordant vs. discordant hrHPV positive specimens. The significance of the differences between proportions used a Z score test. All statistical calculations were performed using SAS v.9.3 (SAS Institute Inc., Cary NC, USA).

## Results

### HC2 and COBAS positivity rates

Among 6,214 women enrolled to the Safety Arm, 10 (0.16 %) and 11 (0.18 %) respectively had invalid HC2 and COBAS results, and 21 (0.34 %) had one or more missing HC2, COBAS or LA results. The present analysis includes the 6,172 (99.3 %) women with valid baseline HC2, COBAS and LA results. Overall HC2/COBAS agreement was 96.1 % (95 % CI: 95.6 – 96.6) and Cohen’s kappa was 0.75 (95 % CI: 0.72 – 0.79) (Table 1). Positive and negative agreements were 77.5 % (95 % CI: 74.7 – 80.3) and 97.9 % (95 % CI: 97.6 – 98.2) respectively. The respective baseline HC2 and COBAS positivity rates were 8.4 % (516/6,172; 95 % CI: 7.7 – 9.1) and 8.8 % (542/6,172; 95 % CI: 8.1 – 9.5). HC2 positivity rates for those 25–29, 30–34, 35–49 and ≥50 years, were 26.6 % (122/458), 15.1 % (84/556), 7.5 % (213/2,859) and 4.2 % (97/2,299) respectively; the corresponding COBAS rates were 27.9 % (128/458), 17.4 % (97/556), 7.7 % (219/2,859) and 4.3 % (98/2,299).

**Table 1:** Digene HC2 and Roche COBAS agreement at baseline

		COBAS								
		All ages			Age <30 year			Age ≥30 year		
		Positive	Negative	Total	Positive	Negative	Total	Positive	Negative	Total
HC2	Positive	410	106	516	108	14	122	302	92	394
	Negative	132	5,524	5,656	20	316	336	112	5,208	5,320
	Total	542	5,630	6,172	128	330	458	414	5,300	5,714
Overall agreement (95 % CI)		96.1 % (95.6, 96.6)			92.6 % (89.7, 94.7)			96.4 % (95.9, 96.9)		
Kappa (95 % CI)		0.75 (0.72, 0.79)			0.81 (0.75, 0.87)			0.73 (0.69, 0.76)		
Positive agreement (95 % CI)		77.5 % (74.7, 80.3)			86.4 % (81.9, 90.9)			74.8 % (71.4, 78.1)		
Negative agreement (95 % CI)		97.9 % (97.6, 98.2)			94.9 % (93.2, 96.6)			98.1 % (97.8, 98.3)		

*Abbreviations*: *HC2* Digene Hybrid Capture® 2 high-risk HPV DNA test®, *COBAS* Roche cobas® 4800 HPV test, *CI* confidence interval

### COBAS genotyping

COBAS genotyping demonstrated 2.1 % (128/6,172) HPV 16 positive, 0.7 % (46/6,171) HPV 18 positive and 7.1 % (437/6,171) OHR HPV positive (Table 2). COBAS and LA overall genotyping agreements ranged from 97.9 to 99.8 %, positive agreements from 85.7 to 87.0 %, and the kappa’s (0.85 – 0.87) indicated excellent genotyping agreement.

**Table 2:** Roche COBAS and linear array genotyping agreement at baseline

Linear array	COBAS														
	HPV 16 *n* = 6,172					HPV 18 *n* = 6,171^a^					Other high risk HPV *n* = 6,171^a^				
	Pos	Neg	Overall agreement (95 % CI)	Kappa (95 % CI)	Pos agreement (95 % CI)	Pos	Neg	Overall agreement (95 % CI)	Kappa (95 % CI)	Pos agreement (95 % CI)	Pos	Neg	Overall agreement (95 % CI)	Kappa (95 % CI)	Pos agreement (95 % CI)
Pos	114	20	99.4 % (99.2 – 99.6)	0.87 (0.82 – 0.91)	87.0 % (82.7 – 91.4)	42	9	99.8 % (99.6 – 99.9)	0.86 (0.79 – 0.94)	86.6 % (79.4 – 93.8)	388	80	97.9 % (97.5 – 98.2)	0.85 (0.82 – 0.87)	85.7 % (83.3 – 88.2)
Neg	14	6,024				4	6,116				49	5,654			

^a^One COBAS HPV 16 positive sample had invalid HPV 18 and Other High Risk HPV results (i.e., no beta-globin detected in the specimen)

*Abbreviations*: *COBAS* Roche cobas® 4800 HPV test, *CI* confidence interval

### HC2 and COBAS result discordance

Discordant HC2/COBAS results were observed among women of all ages, but were more common among those 25–29 years (34/458; 7.4 %) than those 30 years and older (204/5,714; 3.6 %; *p* <0.0001). HC2+/COBAS+ specimens had significantly higher median HC2 RLU/CO values compared to those HC2+/COBAS– (54.9 vs. 5.9; *p* <0.0001). Similarly, HC2+/COBAS+ specimens had significantly lower median COBAS Ct values (30.2 vs. 38.3; *p* <0.0001) than HC2–/COBAS+ specimens. Median Ct values were similar when stratified by COBAS genotype (Table 3). Reflex LBC was abnormal for 36.3 % of those HC2+/COBAS+, 23.6 % of HC2+/COBAS–, 4.5 % of HC2–/COBAS+ and 0.5 % of HC2–/COBAS– (Additional file 1: Table S1).

**Table 3:** RLU/CO and Ct values for concordant and discordant digene HC2 and Roche COBAS results

	RLU/CO	Ct (all)	Ct (HPV 16 only)	Ct (HPV 18 only)	Ct (OHR only)
	Median (Q1, Q3)	Median (Q1, Q3)	Median (Q1, Q3)	Median (Q1, Q3)	Median (Q1, Q3)
HC2+/COBAS+	54.9 (8.6, 368.4)	30.2 (26.6, 34.2)	29.4 (26.6, 34.2)	30.7 (27.2, 35.0)	30.3 (26.3, 33.8)
HC2+/COBAS−	5.9 (2.7, 16.4)	–	–	–	–
HC2−/COBAS+	0.3 (0.2, 0.5)	38.3 (36.8, 39.1)	39.0 (38.0, 40.0)	38.1 (37.0, 38.8)	38.2 (36.7, 38.9)
HC2−/COBAS−	0.1 (0.1, 0.2)	–	–	–	–

*Abbreviations*: *HC2* Digene Hybrid Capture® 2 high-risk HPV DNA test®, *COBAS* Roche cobas® 4800 HPV test, *RLU/CO* relative light unit/cutoff, *Ct* cycle threshold, *Q1* first quartile, Q3 third quartile

### Correlation of HC2 and COBAS results with linear array and reflex LBC

LA confirmed hrHPV genotypes in 96.3 % of HC2+/COBAS+ enrollment specimens (Additional file 1: Table S1). For those with discordant results, HC2+/COBAS− were less likely to contain hrHPV genotypes (12.3 % vs. 68.9 %; *p* <0.0001) and more likely to contain only lrHPV genotypes (52.8 % vs. 12.1 %; *p* <0.0001) than those HC2−/COBAS+. There was no significant difference in the overall abnormal reflex LBC proportions for those with discordant HC2/COBAS results (23.6 % vs. 4.5 %; *p* = 0.47), nor for those discordant specimens containing only lrHPV (26.8 % vs. 18.8 %; *p* = 0.75). Of interest, specimens containing multiple hrHPV (43 % vs. 28 %; *p* = 0.002) and lrHPV (42 % vs. 9 %; *p* = 0.06) genotypes by LA were more likely to have abnormal LBC compared to those with a single hr- or lrHPV genotype (Additional file 1: Table S2).

### CIN detection

Overall round 1 and 2 CIN2 and CIN3+ rates classified by HC2 and COBAS results are shown in Table 4. At round 1, the respective rates were 8.6/1,000 (95 % CI: 6.3 – 10.9) and 6.6/1,000 (95 % CI: 4.6 – 8.7), whereas at round 2 (LBC screening, with HC2 triage of ASCUS), the overall rates were 0.7/1,000 (95 % CI: 0.0 – 1.4) and 0.4/1,000 (95 % CI: 0.0 – 0.9) respectively.

**Table 4:** Rounds 1 and 2 pathology stratified by baseline Digene HC2 and Roche COBAS results

Baseline HC2/COBAS	Round 1 (Baseline + 12 months follow-up)							Round 2 (24 months)						
	Cytology	N	Colpo Referred/Completed	CIN2		CIN3+		Cytology	N	Colpo Referred/Completed	CIN2		CIN3+	
				N	Rate^a^ (95 % CI)	N	Rate^a^ (95 % CI)				N	Rate^a^ (95 % CI)	N	Rate^a^ (95 % CI)
Pos/Pos	UNSAT	1	1/1					UNSAT	1	0/0				
	NILM	260	152/143	22		16		NILM	208	0/0				
	ASCUS	35	35/33	6		2		ASCUS	4	3/3				
	LSIL	68	68/63	9		4		LSIL	6	6/6				
	ASCH	18	18/15	4		5		ASCH	2	2/2				
	HSIL	28	28/25	7		13		HSIL	1	1/1				
	Total	410	302/280	48	7.8 (5.6 – 10.0)	40	6.5 (4.5 – 8.5)	Total	222	13/13	0	–	0	–
Pos/Neg	UNSAT	0	0/0					UNSAT	1	0/0				
	NILM	81	27/23	4				NILM	75	0/0				
	ASCUS	10	10/9	1				ASCUS	2	1/1				
	LSIL	13	13/12					LSIL	4	4/4				
	ASCH	2	2/2			1		ASCH	0	0/0				
	HSIL	0	0/0					HSIL	0	0/0				
	Total	106	52/46	5^b^	0.8 (0.1 – 1.5)	1^c^	0.2 (0.0 – 0.5)	Total	82	5/5	0	–	0	–
Neg/Pos	UNSAT	3						UNSAT	1	0/0				
	NILM	123						NILM	117	0/0				
	ASCUS	1						ASCUS	0	0/0				
	LSIL	5						LSIL	2	2/2				
	ASCH	0						ASCH	0	0/0				
	HSIL	0						HSIL	1	1/1	1			
	Total	132						Total	121	3/3	1^d^	0.2 (0.0 – 0.5)	0	-
Neg/Neg	UNSAT	12						UNSAT	24	6/5				
	Not done	4901												
	NILM	585						NILM	4957	1/0				
	ASCUS	17						ASCUS	31	2/2				
	Other atypia	0						Other atypia	6	6/6				
	LSIL	8						LSIL	38	38/36	2			
	ASCH	1						ASCH	4	4/4				
	HSIL	0						HSIL	5	5/5	1		2	
	Total	5524						Total	5065	62/58	3^e^	0.6 (0.0 – 1.3)	2^f^	0.4 (0.0 – 0.9)
Total		6172	354/326	53	8.6 (6.3 – 10.9)	41	6.6 (4.6 – 8.7)	Total	5490	83/79	4	0.7 (0.0 – 1.4)	2	0.4 (0.0 – 0.9)

^a^Rate per 1,000 women screened. ^b^By Linear Array, two were HPV 52 positive, one HPV 56 positive, one HPV 66 positive and one HPV negative. ^c^Linear Array was HPV negative; a LEEP one month later showed CIN2. ^d^Linear Array was HPV 16 and HPV 51 positive at both baseline and 24 months. ^e^By Linear Array, two were HPV negative at baseline; at 24 months one was HPV 66 positive and the second was HPV 35 and HPV 58 positive. The third case was HPV 66 positive at baseline and HPV 16, HPV 18 and HPV 66 positive at 24 months. ^f^By Linear Array, one was HPV 16 and HPV 52 positive at baseline and HPV 52 positive at 24 months; the second was HPV 16 positive at both baseline and 24 months

*Abbreviations*: *HC2* Digene Hybrid Capture® 2 high-risk HPV DNA test®, *COBAS* Roche cobas® 4800 HPV test, *UNSAT* smear unsatisfactory; *NILM* negative for intraepithelial lesions and malignancy, *ASCUS* atypical squamous cells undetermined significance, *ASCH* atypical squamous cells, cannot rule out high-grade, *LSIL* low-grade squamous intraepithelial lesion, *HSIL* high-grade squamous intraepithelial lesion, *CIN* cervical intraepithelial neoplasia, *LEEP* loop electrosurgical excision procedure

Of the round 1 CIN2 and CIN3+, 48/53 (90.6 %) and 40/41 (97.6 %) respectively were concordant HC2+/COBAS+. The CIN3 identified among HC2+/COBAS− discordant subjects was HPV negative by LA. This subject underwent a loop electrosurgical excision procedure (LEEP) 1 month following the biopsy, at which time CIN2 was identified, suggesting that this lesion may have been destined to regress spontaneously. Immunohistochemistry for p16 was not performed on either the biopsy or the LEEP specimen. Due to the study blinding, it was not possible to obtain histopathology at round 1 for discordant HC2−/COBAS+ subjects, who were not referred to colposcopy unless they had abnormal LBC at round 2, 24 months later. At the 24 month exit LBC screen, among the enrollment HC2−/COBAS+ subjects, one CIN2 was identified, and at baseline, this subject was HPV 16 and HPV 51 positive by both COBAS and LA with NILM LBC. At round 2, LBC had progressed to high-grade squamous intraepithelial lesion and the subject was still HPV 16 and HPV 51 positive by both COBAS and LA. Of the two CIN3 at round 2 among enrollment HC2−/COBAS− subjects, one was LA HPV 16 and HPV 52 positive at enrollment and HPV 52 positive at exit; the second was HPV 16 positive at both enrollment and exit. This suggests that these lesions may have been present at baseline but were not detected by either HC2 or COBAS.

Of the 94 CIN2+ identified in Round 1, 52 (55 %) had LBC ≥ASCUS at baseline; of the remaining subjects with NILM LBC at baseline, 16 (17 %) were HPV 16 or 18 positive. Therefore, if both abnormal LBC and presence of HPV 16/18 genotypes had been used for colposcopy referral, 68/94 (72 %) CIN2+ cases would likely have been identified at the baseline screen, with the remainder identified when 12 month follow-up testing indicated persistent non-HPV 16/18 infection.

Regular audits of the provincial registry for CIN2+ lesions that were not based on a FOCAL trial colposcopy referral identified one adenocarcinoma and one endometrial cancer, both diagnosed ~20 months after the baseline screens (HC2, COBAS and LA were negative for both cases). Both subjects underwent hysterectomy and exited the trial.

### HC2 clearance and CIN outcomes

**Fig. 2:**
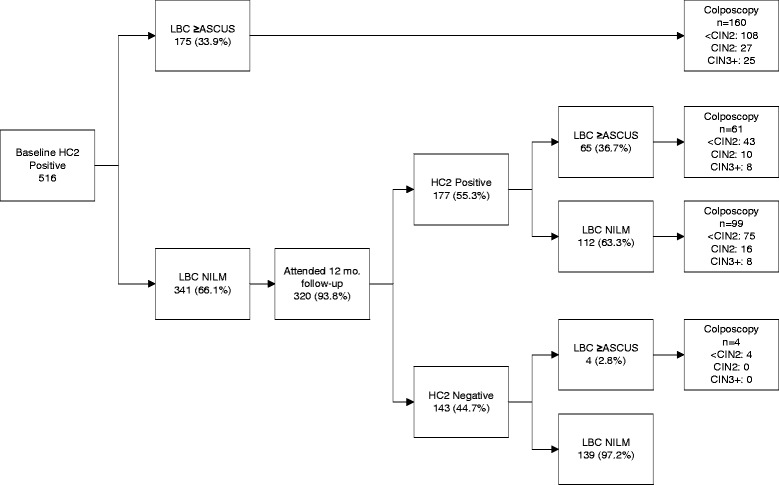
Round 1 CIN Outcomes and HPV Clearance Rates for Baseline Digene HC2 Positive Subjects. *Abbreviations*: *HC2* Digene Hybrid Capture® 2 high-risk HPV DNA test®, *LBC* liquid-based cytology, *ASCUS* atypical squamous cells, undetermined significance, *NILM* negative for intraepithelial lesions and malignancy

Figure 2 illustrates the round 1 HPV clearance rates and CIN outcomes for the 516 baseline HC2 positive women. Of those HC2+/LBC NILM who attended the 12 month follow-up screen, 177/320 (55.3 %) were persistently HC2 positive, among whom 26/53 (49.1 %) of the round 1 CIN2 and 16/41 (39.0 %) of the round 1 CIN3+ were identified. LBC progression from NILM at baseline to ≥ASCUS at 12 months was not associated with a higher likelihood of having CIN2+ vs. those without LBC progression. There was no CIN2+ among the 143 women who cleared their hrHPV infections by 12 months.

### Colposcopy triage by reflex LBC and HPV genotyping

Since HC2−/COBAS+ women were not referred to colposcopy at round 1, assessment of the contributions of HPV genotype and reflex LBC to CIN2 and CIN3+ outcomes was performed only for HC2+/COBAS+ subjects (*n* = 410). For CIN2, by COBAS, 11/48 (23 %) were HPV 16 positive; 7/48 (15 %) were HPV 18 positive; and 32/48 (67 %) were positive only for non-HPV 16/18 OHR HPV. For CIN2, there were no significant differences based on HPV genotype (*p* = 0.60) (Table 5). For CIN3+, 23/40 (58 %) were HPV 16 positive, 6/40 (15 %) were HPV 18 positive and 13/40 (33 %) were positive only for non-HPV 16/18 OHR HPV. HPV 16 positive subjects were significantly more likely to have CIN3+ compared to those positive for non-HPV 16 genotypes (odds ratio [OR] 5.11, 95 % CI 2.30, 11.37; *p* = 0.0003), but there was no significant difference for HPV 18 positive vs. non-HPV 16/18 OHR positive subjects (OR 2.62; 95 % CI 0.73, 9.49). HPV 16 positive subjects had a higher proportion of CIN3+ (58 % [23/41]) vs. 23 % [11/48] for CIN2), whereas the proportions were the same for HPV 18 (15 % [7/48] vs. 15 % [6/40]). Conversely, subjects positive only for non-HPV 16/18 OHR HPV had a higher CIN2 proportion compared to CIN3+ (67 % [32/48] vs. 33 % [13/40]).

**Table 5:** Multivariate analysis for association of HPV genotype and reflex LBC with CIN2 and CIN3+

	CIN2		CIN3+	
	*p* value	Odds ratio (95 % CI)	*p* value	Odds ratio (95 % CI)
HPV genotype	0.60		**0.0003**	
16 vs. non-16 OHR		0.71 (0.33, 1.53)		**5.11 (2.30, 11.37)**
18 vs. non-16/18 OHR		1.21 (0.42, 3.50)		2.62 (0.73, 9.49)
Reflex LBC	0.15		**<0.0001**	
High-grade^a^ vs. NILM		2.23 (0.96, 5.17)		**5.99 (2.53, 14.18)**
Low-grade^b^ vs. NILM		1.02 (0.50, 2.08)		0.54 (0.20, 1.49)

^a^High-grade = ASCH + HSIL. ^b^Low-grade = ASCUS + LSIL

*Abbreviations*: *OHR* other high-risk HPV, *LBC* liquid-based cytology, *NILM* negative for intraepithelial lesions and malignancy, *CIN* cervical intraepithelial neoplasia, *ASCUS* atypical squamous cells undetermined significance, *ASCH* atypical squamous cells, cannot rule out high-grade; *LSIL* low-grade squamous intraepithelial lesion, *HSIL* high-grade squamous intraepithelial lesion

Bolded type indicates statistically significant results

For reflex LBC, those with high-grade vs. NILM LBC were significantly more likely to have CIN3+ (OR 5.99, 95 % CI 2.53, 14.18; *p* <0.0001) compared to those with low-grade vs. NILM LBC. In contrast, there was no significant difference for CIN2 for those with either high-grade or low-grade vs. NILM LBC (*p* = 0.15). There were no significant interactions between HPV genotype and LBC (data not shown).

## Discussion

In this sub-study of the HPV FOCAL Trial Safety Arm, we demonstrated that over a 2 year screening interval with hrHPV screening at baseline followed by LBC screening at 24 months, hrHPV testing at baseline identified 94 % of the CIN2+ lesions detected over the two screening rounds. Very low CIN2+ rates were observed at the exit LBC screen, which supports the safety of hrHPV vs. LBC screening every 2 years. It is important to note that in this study women were managed only on their HC2 and corresponding cytology results (COBAS results were blinded). Despite the occurrence of discordant baseline HC2 and COBAS results, 43/44 round 1 CIN3+ were among those who were both HC2 and COBAS positive. If women had been managed on both HC2 and COBAS results, it is possible that additional CIN lesions might have been identified at baseline among those HC2−/COBAS+. In agreement with our findings, a comparison study by Cuzick et al. [7] of the performance of six HPV assays in a population referred to colposcopy on the basis of abnormal cytology, showed that both HC2 and COBAS were positive for all CIN3+ cases. However, in that study HPV positive/cytology normal women, among whom greater discordance between various HPV assays and CIN detection might be expected, were not referred to colposcopy. The full impact of discordant HPV results among cytologically normal women remains under investigation. It is not unexpected that a given hrHPV assay may miss a potentially significant CIN, as other trials, including ALTS, ARTISTIC and POBASCAM, noted that a proportion of CIN2+ test negative for hrHPV [20–22]. Of note, co-testing would have identified the CIN3+ that was not detected by COBAS at baseline, since the LBC result was atypical squamous cells, cannot rule out high-grade, which would have resulted in a colposcopy referral. Some studies have shown that co-testing has a higher sensitivity for CIN2+ than hrHPV screening alone [23, 24], whereas others have not demonstrated a difference [25, 26].

The round 1 CIN2 and CIN3+ rates based on primary hrHPV screening (8.6/1,000 and 6.6/1,000 respectively) were higher than in the HPV FOCAL Control Arm (LBC screening, with HC2 triage of ASCUS) which had round 1 rates of 6.0/1,000 and 5.0/1,000 respectively [27]. The CIN2+ rate in this study (15.2/1,000) was also higher than the 2011 CIN2+ rate of 6.3/1,000 for women aged 20–69 in the BC provincial cervical screening program (using conventional cytology) [28]. The round 1 CIN rates in our study are similar to the verification bias-adjusted rates in the ATHENA trial (CIN2: 8.6 vs. 8.0; CIN3+: 6.6 vs. 10.0) [29]. The FOCAL trial did not adjust for verification bias, but there are some differences in the study populations, e.g., the mean age of FOCAL vs. ATHENA subjects (46 vs. 40 years) and those who ever smoked (36.4 % vs. 29.3 %). The FOCAL trial had a higher proportion of individuals with Asian ethnic background (12.5 % vs. 1.6 %), whereas ATHENA had a higher proportion of Black/African American individuals (14.0 %) compared to FOCAL (3.3 %, which includes Aboriginals) [27, 29].

While our study had a lower COBAS positivity rate than ATHENA (8.8 % vs. 12.6 %) [29], in both studies HPV 16 (2.1 % vs. 2.8 %) was approximately three times more commonly detected than HPV 18 (0.7 % vs. 1.0 %). These data are consistent with other studies [7], including a recent BC population-based HPV prevalence survey among unvaccinated women aged 15–69 years attending cervical screening (HPV 16: 2.7 %; HPV 18: 1.1 %, using LA) [30]. Our study identified similar HPV 16- but higher HPV 18-attributable CIN2 and CIN3+ proportions compared to ATHENA. We identified HPV 16 in 23 % (11/48) of CIN2 vs. 30 % (57/192) for ATHENA, and 58 % (23/40) vs. 50 % (153/305) for CIN3+. We identified HPV 18 in 15 % (7/40) of CIN2 compared to 3 % (6/192) for ATHENA, and 15 % (6/40) vs. 8 % (25/305) for CIN3+.

For concordant HC2/COBAS positive subjects, CIN3+ was significantly more likely when LBC was high-grade vs. NILM. Reflex LBC dysplasia was not significantly different for CIN2. CIN3+ was significantly more likely for HPV 16 vs. non-HPV 16 HPV positive subjects, while no significant differences in CIN3+ were found for HPV 18 vs. non-HPV 16/18 OHR genotypes. There was no significant difference between the CIN2-associated HPV genotypes. These findings are in agreement with the ATHENA trial which concluded that a combination of HPV 16/18 genotyping and reflex cytology is a suitable triage strategy for hrHPV positive women [26].

Specimens with discordant HC2/COBAS results had RLU/CO ratios and Ct values significantly closer to the test limits of detection than concordant positive specimens. It seems reasonable that specimens likely to have lower viral loads (based on low RLU/CO ratios or high Ct values) would be more likely to produce discordant results with different assays. Furthermore, some of these discordant results likely reflect false-positives [31], which are less likely to be consistent between assays. This is supported by the lower CIN2+ rates among women with HC2+/COBAS− results.

Our finding that 45 % of HC2 positive women with NILM LBC at baseline cleared their hrHPV infections after 12 months is consistent with the published literature [11, 32, 33]. The high rate of HPV clearance supports recommendations that when LBC is used for triage, an appropriate way to manage hrHPV positive women with NILM cytology is by re-testing for HPV clearance after a short follow-up period, e.g., 12–18 months [33, 34]. However, this may result in a high rate of loss to follow-up of these women, some of whom will have underlying CIN2+ [9, 20, 26]. A triage test which efficiently identifies a high likelihood of CIN2+ at the time of the initial positive HPV screen test would be of considerable benefit.

Specimens containing only lrHPV types frequently had low-grade LBC abnormalities [11 % (57/516) of HC2 and 3 % (17/542) of COBAS positive results] which may have implications for selection of a primary HPV screening assay if LBC is to be used for triage. Since lrHPV’s have a negligible association with CIN2+ [35], these individuals would not benefit from colposcopy referral. Full genotyping of all hrHPV positives will become increasingly feasible with the availability of automated commercial genotyping assays and this information could be used to prioritize women for colposcopy. However, cost considerations may preclude its use to help identify women with false positive hrHPV screening results. This emphasizes the need for better triage tests to avoid colposcopy over-referral and the associated potential harms.

An important limitation of this study was the inability to obtain CIN outcomes at round 1 for women with HC2−/COBAS+ discordant results, since baseline COBAS results were blinded until after study exit. This precluded comparison of the sensitivity and specificity of the HC2 and COBAS assays. In addition, had these women been referred to colposcopy at baseline, it is possible that additional CIN lesions would have been detected, as was observed for the HC2+/COBAS− group. Furthermore, no hrHPV testing was performed at the 24 month study exit except for HC2 triage of women with ASCUS LBC. Although one CIN2 was identified at round 2 among baseline HC2−/COBAS+ women, it is possible that the use of only LBC at round 2 was not sufficiently sensitive to demonstrate whether this group of women had underlying CIN lesions not detected by HC2 at baseline. A future analysis of the FOCAL Intervention Arm where women were screened with HC2 and COBAS at both enrollment and the 48 month exit screen may provide further insight. However, Intervention Arm COBAS enrollment results were also blinded and not used for subject management, but both HC2 and COBAS were used for colposcopy referral at the 48 month exit screen. Furthermore, there is an opportunity to follow these women for significant CIN outcomes over longer time periods once they exit the trial and return to the centralized cervical screening program. Long term follow-up of Swedescreen subjects after a baseline hrHPV positive screen showed that incidence rate ratios for CIN2+ remained high for greater than nine years [36], especially for those with HPV 16, 18, 31 and 33 identified at baseline.

Another limitation relates to the retrospective cytology interpretations. Reflex LBC for HC2 positive women was performed as it would be in a real setting, whereas LBC slides for HC2 negative, COBAS and/or LA positive women were examined approximately two years later. This could have biased the LBC interpretations relative to those conducted in real-time.

## Conclusions

This study demonstrated that over a 2 year screening interval with hrHPV testing at baseline, followed by LBC screening at 24 months, 94 % of CIN2+ detected over the two screening rounds were hrHPV positive at baseline. Women were managed at round 1 only on their HC2 and corresponding LBC results, but 43/44 CIN3+ were detected among those HC2+/COBAS+. It is possible that additional CIN cases would have been detected among HC2−/COBAS+ subjects if COBAS results had also been used for subject management. Of the CIN2+ identified in Round 1, 55 % had abnormal cytology and an additional 17 % were HPV 16 or 18 positive at baseline, demonstrating the added benefit of immediate referral of women with these genotypes to potentially identify CIN2+ earlier than by follow-up testing of those with NILM cytology. Therefore, the results of this study support the use of reflex LBC together with HPV16/18 genotyping for colposcopy triage of hrHPV positive women.  However, as the cohort of HPV vaccinated women reaches cervical screening age, the benefits of HPV 16/18 genotyping for triage will be diminished.

## Additional file

Additional file 1:
**Table S1.** Baseline Digene HC2/Roche COBAS Results Stratified by LA and Cytology. **Table S2**. Baseline Digene HC2 and Roche COBAS Abnormal Cytology Rates Stratified by Linear Array Genotyping. (DOCX 16 kb)

## Abbreviations

ASCUS: Atypical squamous cells, undetermined significance
BC: British Columbia
CI: Confidence interval
CIN: Cervical intraepithelial neoplasia
COBAS: Roche cobas® 4800 HPV test
Ct: Cycle threshold
HC2: Digene Hybrid Capture® 2 HPV test
HPV: Human papillomavirus
hr: High-risk
LA: Roche linear array HPV genotyping test
LBC: Liquid-based cytology
LEEP: Loop electrosurgical excision procedure
lr: Low-risk
NILM: Negative for intraepithelial lesions or malignancy
OHR: Other high-risk
OR: Odds ratio
Q1: First quartile
Q3: Third quartile
RLU/CO: Relative light units/cutoff
