# Supplementary material for: Comparison of the Roche cobas® 4800 and Digene Hybrid Capture® 2 HPV tests for primary cervical cancer screening in the HPV FOCAL trial
Source: BMC Cancer. 2015 Dec 16;15:968. doi: 10.1186/s12885-015-1959-5 (PMC4682219; doi:10.1186/s12885-015-1959-5)
Supplement: Additional file 1: — Table S1. Baseline Digene HC2/Roche COBAS Results Stratified by LA and Cytology. Table S2. Baseline Digene HC2 and Roche COBAS Abnormal Cytology Rates Stratified by Linear Array Genotyping. (DOCX 16 kb) [file 12885_2015_1959_MOESM1_ESM.docx]

Additional file 1: Table S1. Baseline Digene HC2/Roche COBAS Results Stratified by LA and Cytology

| HC2/COBAS | Linear Array | N | % | Cytology | | | | | | | |
| --- | --- | --- | --- | --- | --- | --- | --- | --- | --- | --- | --- |
|  |  |  |  | Not done | UNSAT | NILM | ASCUS | LSIL | ASCH | HSIL | % ≥ASCUS |
| Pos/Pos | Pos hr^a^ | 395 | 96.3 |  | 1 | 246 | 35 | 68 | 18 | 27 | 37.5 |
|  | Pos lr only | 1 | 0.2 |  |  | 1 |  |  |  |  | 0.0 |
|  | Neg | 14 | 3.4 |  |  | 13 |  |  |  | 1 | 7.1 |
|  | Total | 410 |  | 0 | 1 | 260 | 35 | 68 | 18 | 28 | 36.3 |
|  | | | | | | | | | | | |
| Pos/Neg | Pos hr^a^ | 13 | 12.3 |  |  | 8 | 2 | 2 | 1 |  | 38.5 |
|  | Pos lr only | 56 | 52.8 |  |  | 41 | 4 | 11 |  |  | 26.8 |
|  | Neg | 37 | 34.9 |  |  | 32 | 4 |  | 1 |  | 13.5 |
|  | Total | 106 |  | 0 | 0 | 81 | 10 | 13 | 2 | 0 | 23.6 |
|  | | | | | | | | | | | |
| Neg/Pos | Pos hr^a^ | 91 | 68.9 |  | 3 | 85 | 1 | 2 |  |  | 3.3 |
|  | Pos lr only | 16 | 12.1 |  |  | 13 |  | 3 |  |  | 18.8 |
|  | Neg | 25 | 18.9 |  |  | 25 |  |  |  |  | 0.0 |
|  | Total | 132 |  | 0 | 3 | 123 | 1 | 5 | 0 | 0 | 4.5 |
|  | | | | | | | | | | | |
| Neg/Neg | Pos hr^a^ | 92 | 1.7 |  | 1 | 86 | 2 | 3 |  |  | 5.4 |
|  | Pos lr only | 529 | 9.6 |  | 10 | 498 | 15 | 5 | 1 |  | 4.0 |
|  | Neg | 4903 | 88.8 | 4901 | 1 | 1 |  |  |  |  | 0.0 |
|  | Total | 5524 |  | 4901 | 12 | 585 | 17 | 8 | 1 | 0 | 0.5 |

^a^Specimen is positive for at least one high-risk HPV genotype but may also be positive for low-risk HPV genotypes.

Abbreviations: hr: high-risk; lr: low-risk; UNSAT: smear unsatisfactory; NILM: negative for intraepithelial lesions and malignancy; ASCUS: atypical squamous cells undetermined significance; LSIL: low-grade squamous intraepithelial lesion; ASCH: atypical squamous cells, cannot rule out high-grade; HSIL: high-grade squamous intraepithelial lesion

Additional file 1: Table S2. Baseline Digene HC2 and Roche COBAS Abnormal Cytology Rates Stratified by Linear Array Genotyping

| Linear Array | HC2 Positive (n=516) | | | COBAS Positive (n=542) | | | HC2 and/or COBAS Positive (n=648) | | | P value  (proportion ≥ASCUS) |
| --- | --- | --- | --- | --- | --- | --- | --- | --- | --- | --- |
|  | n | ≥ASCUS (n) | % | n | ≥ASCUS (n) | % | n | ≥ASCUS (n) | % |  |
| Pos hr HPV^a^ | 408 | 153 | 38% | 486 | 151 | 31% | 499 | 156 | 31% |  |
| Pos hr HPV^a^  (one genotype) |  |  |  |  |  |  | 388 | 108 | 28% | **0.002** |
| Pos hr HPV^a^  (≥ two genotypes) |  |  |  |  |  |  | 111 | 48 | 43% |  |
| Pos lr HPV only | 57 | 15 | 26% | 17 | 3 | 18% | 73 | 18 | 25% |  |
| Pos lr HPV only (one genotype) |  |  |  |  |  |  | 54 | 10 | 19% | 0.06 |
| Pos lr HPV only  (≥ two genotypes) |  |  |  |  |  |  | 19 | 8 | 42% |  |
| Negative | 51 | 6 | 12% | 39 | 1 | 3% | 76 | 6 | 8% |  |

^a^Specimen is positive for at least one high-risk HPV genotype but may also be positive for low-risk HPV genotypes

Abbreviations: HC2: Digene Hybrid Capture® 2 High-Risk HPV DNA Test®; COBAS: Roche cobas® 4800 HPV Test; hr: high-risk; lr: low-risk; ASCUS: atypical squamous cells undetermined significance
